# Co-culture of *Bacillus coagulans* and *Candida utilis* efficiently treats *Lactobacillus* fermentation wastewater

**DOI:** 10.1186/s13568-019-0743-3

**Published:** 2019-01-30

**Authors:** Jiyun Liu, Peifu Shi, Shahbaz Ahmad, Chunhua Yin, Xiaolu Liu, Yang Liu, Haiyang Zhang, Qianqian Xu, Hai Yan, Qingxiao Li

**Affiliations:** 1 School of Chemistry and Biological Engineering, University of Science and Technology, Beijing, Beijing, 100083 People’s Republic of China; 2 College of Tropical Agriculture and Human Resources, University of Hawaii, Honolulu, HI 96822 USA

**Keywords:** *Bacillus coagulans*, *Candida utilis*, Co-culture, *Lactobacillus* fermentation wastewater, Treatment

## Abstract

Co-culture of *Bacillus coagulans* and *Candida utilis* was firstly investigated in the efficient treatment of *Lactobacillus* fermentation wastewater (LFW) containing total organic carbon (TOC) of 22.0 g/L and total nitrogen (TN) of 2.4 g/L. The utilization of lactic acid by *C. utilis* was responsible for the relief of feedback inhibition to promote the growth of *B. coagulans.* The removal ratio of TOC by *B. coagulans* and *C. utilis* was only 9.1% and 22.7%, respectively, which was improved to 49.0% by co-culture. The removal ratio of TN by *B. coagulans* and *C. utilis* was merely 6.3% and 12.5%, respectively, which was also promoted to 44.6% by co-culture. Both the high growth of *B. coagulans* and the efficient removal of TOC and TN from LFW was achieved with the co-culture, which is not previously reported and very important in the production of probiotics with the resource utilization of LFW.

## Introduction

Lactic acid bacteria (LAB) are widely distributed in nature and are of great importance and value in agriculture, animal husbandry, food, medicine and manufacturing industries (Leroy and De Vuyst 2014). LAB in the animal body carry out a variety of physiological functions such as regulating normal gastrointestinal tract flora, maintaining micro-ecological balance, and inhibiting the growth of intestinal pathogenic bacteria, which improves the body immunity (Jahn et al. 1996). Mattia Pia Arena provided a fullest possible overview of the antiviral and antifungal activities ascribed to probiotic LAB (Arena et al. 2018). Zeng et al. (2017) and Mao et al. (2017) used LAB as a safe and convenient oral delivery of bioactive peptide and protein drugs for the treatment of diabetes. Furthermore, LAB is commonly used in the manufacture of yoghurt, cheese, sauerkraut (Zhao et al. 2016) and other fermented food (Di Cagno et al. 2016). With the increasing need for LAB, both their culturing scale and the production of *Lactobacillus* fermentation wastewater (LFW) are rapidly growing. LFW is generally rich with organic compounds such as sugars and protein, as well as lactic acid produced by LAB, which causes pollution and eutrophication in natural water bodies when it is directly released into the environment. Unfortunately, the treatment technology of LFW, especially recycling it as a culture media for other probiotic strains, has not been reported in the literature up to now.

As a typical bacterial probiotic strain, *Bacillus coagulans* can produce lactic acid and has an ability in the sporulation to overcome harsh stresses, which can inhibit the growth of intestinal pathogens and regulate the animal intestinal micro-ecological balance (Riazi et al. 2009, 2012), and promote animal digestion, and improve animal immunity (Grata and Nabrdalk 2012; Kodali and Sen 2008). Choi et al. (2016) reported that the mixed-culture is a viable approach for the economically feasible production of lactic acid due to the potential use of organic waste for *B. coagulans* as feedstock.

*Candida utilis* is another typical eukaryotic probiotic strain that can produce edible protein from various wastes, including bamboo wastewater (Li et al. 2009), rice bran (Rajoka et al. 2004), molasses (Lee and Kim 2001) and potato starch wastewater (Gélinas and Barrett 2007). *C. utilis* can utilize five-carbon, six-carbon sugars and extracellular organic acids, lactic acid (Oliva and Hang 1979), fumarate, and l-malic acid (Saayman et al. 2000), as carbon sources. In addition, as *C. utilis* cells are rich in vitamin B and proteins, they can act as a nutrient supplement for livestock, to improve the digestibility of the feed, maintain the microbial balance of the gastrointestinal tract, and enhance the immunity of the animals. Although the individual pure culture of *B. coagulans* or *C. utilis* has been well investigated (Yadav and Tarafdar 2012), but no information on the co-culture of them has been reported.

Here the co-culture of *B. coagulans* and *C. utilis* using LFW as a medium was investigated, which indicated that the utilization of lactic acid by *C. utilis* was responsible for the relief of feedback inhibition to promote the growth of *B. coagulans.* The removal ratios of both total organic carbon (TOC) and total nitrogen (TN) from LFW were all much improved in the presence of *B. coagulans* and *C. utilis* simultaneously. The aim in this study is to produce probiotics by the efficient resource utilization of nutrients in LFW, which is not mentioned in the literature and play a vital role in environmental science and biotechnology.

## Materials and methods

### Microbial strains and culture conditions

*Bacillus coagulans* (ATCC 7050) and *C. utilis* (ATCC 3052) were bought from Institute of Microbiology, Chinese Academy of Sciences. LFW (Hebei Yiran Biotechnology Co., Ltd.) of *Bifidobacterium bifidum* was centrifuged (10,000×*g* for 10 min), filtered (0.22 μ) and sterilized at 121 °C for 20 min, and the initial pH was adjusted to 7.2 by using 40 g/L NaOH solution. *B. coagulans* grown on beef peptone medium (Chandra et al. 2007) and *C. utilis* grown on Yeast standard medium (Niu et al. 2011) were used as seeds, inoculated into the pretreated LFW and cultured at 35 °C with the shaking rate of 200 r/min for 4 days. The experiments were carried out in the 100 mL Erlenmeyer flasks with 20 mL culture volume. l.0 mL culture solution of *B. coagulans* (cell density 4.67 × 10^7^/mL) or *C. utilis* (cell density 1.53 × 10^7^/mL) was used as inoculum. The samples of three bottles were taken to determine pH, lactic acid, TOC, TN and cell density everyday, respectively.

### Identification and count of cell density with flow cytometry

The total biomass of both *B. coagulans* and *C. utilis* was measured by a spectrophotometer (722 s, China) at a wavelength of 680 nm (OD680 nm). A flow cytometry (Partec CY-S-3001, German) was further used to identify and count the different cell densities of *B. coagulans* and *C. utilis,* respectively. The properties measured include a particle’s relative size, relative granularity or internal complexity, and the number of the particle. For flow cytometer measuring, the gain numeric of handle system parameter was set as follows: FSC 155, SSC 290, FL1 248, FL2 361.5. Suspended cells from 0.2 to 150 μ in size was kept by sieving. 10 μL of sample was diluted with 990 μL phosphate buffer solution (PBS; pH 7.0) to 100 fold, and 800 μL diluted sample was prepared in 2 mL transparent test tubes for a flow cytometer. All samples were measured with the excitation and emission wavelengths of 355 nm at room temperature. Each sample was pumped at 2 m/s for about 1.5 min.

### Determination of pH, TOC, TN and total phosphorus (TP)

A pH meter (PHS-25, Leici, China) was directly used to measure pH of the culture sample. TOC and TN in culture solution that was centrifuged (10,000×*g* for 5 min) and filtered (0.22 μm) with the dilution of ultrapure water were determined on a TOC-V CPH (CPH-CPN, Shimadzu, Japan). TP in LFW was also measured by phosphomolybdenum blue spectrophotometry (Li and Liu 2006).

### Determination of lactic acid

An amount of 2 mL of culture broth was centrifuged at 12,000×*g* for 10 min and the supernatant of 200 μL was taken and diluted 20 times with ultrapure water to measure the concentration of lactic acid on a HPLC system (Shimadzu LC-10A TVP, Shimadzu Co., Ltd., Japan) at the wavelength of 210 nm (De Baere et al. 2013). The mobile phase was 99% (v/v) 0.02 mol/L potassium dihydrogen phosphate solution (adjusted pH to 2 with phosphoric acid) and 1% (v/v) acetonitrile using the column of Agilent ZORBAX SB-Aq (150 mm × 4.6 mm, 5 μm) at 35 °C. The flow rate was 1 mL/min and the injection volume was 20 μL.

## Results

### Main physicochemical indexes in LFW

As shown in Table 1, TOC, TN and TP of LFW in the absence of LAB are 22.1 g/L, 2.4 g/L and 28.5 mg/L, respectively. Furthermore, lower pH 3.8 of LFW may be caused by the production of organic acids such as lactic acid by LAB.

**Table 1:** Physicochemical indexes of LFW

Parameters	Value
TOC (g/L)	22.1
TN (g/L)	2.4
TP (mg/L)	28.5
pH	3.8

### Identification and determination of cell densities of *B. coagulans* and *C. utilis* with flow cytometer

**Fig. 1:**
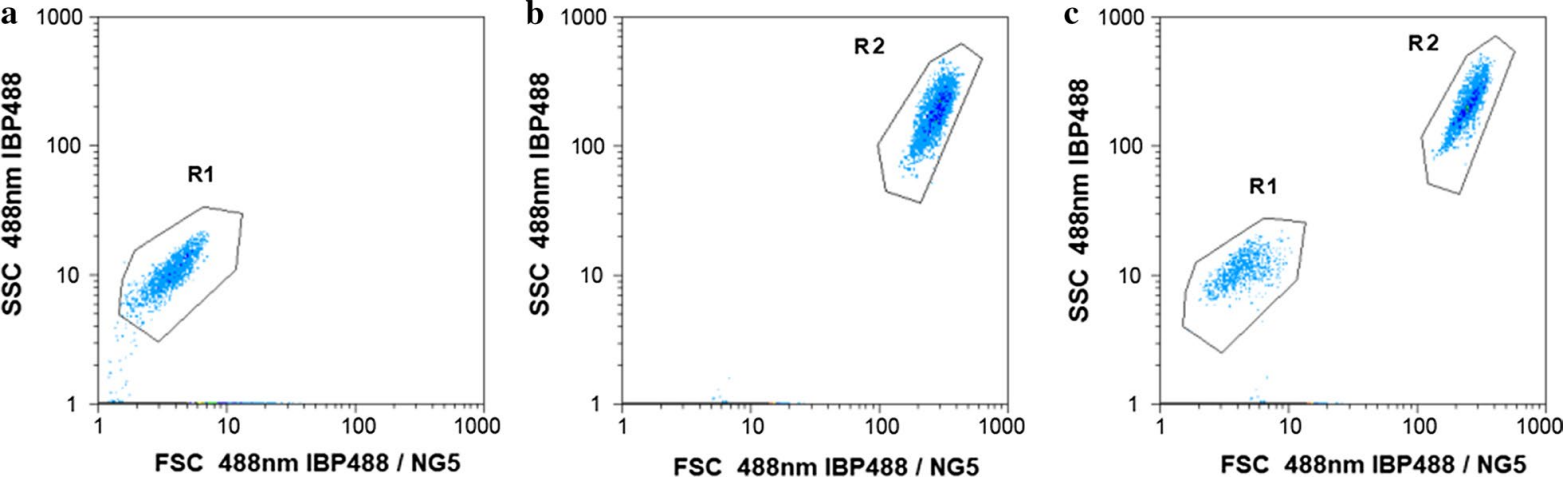
Identification of *B. coagulans* and *C. utilis* with flow cytometer. **a** *B. coagulans.* **b** *C. utilis*. **c** Both *B. coagulans* and *C. utilis*

Figure 1 shows the cell scatter diagram of flow cytometer with FSC as the abscissa and SSC as the ordinate, which indicated that the scatter distribution areas of *B. coagulans* (R1) and *C. utilis* (R2) were concentrated on the lower left (Fig. 1a) and upper right (Fig. 1b) of the coordinate system, respectively. The cell scatter diagram in the presence of both *B. coagulans* (R1) and *C. utilis* (R2) (Fig. 1c) showed that the cells of *B. coagulans* and *C. utilis* could be clearly separated and identified with the different cell size, and their cell numbers could also accurately count with flow cytometer, respectively.

### The growth of *B. coagulans* and *C. utilis* using LFW as culture medium

**Fig. 2:**
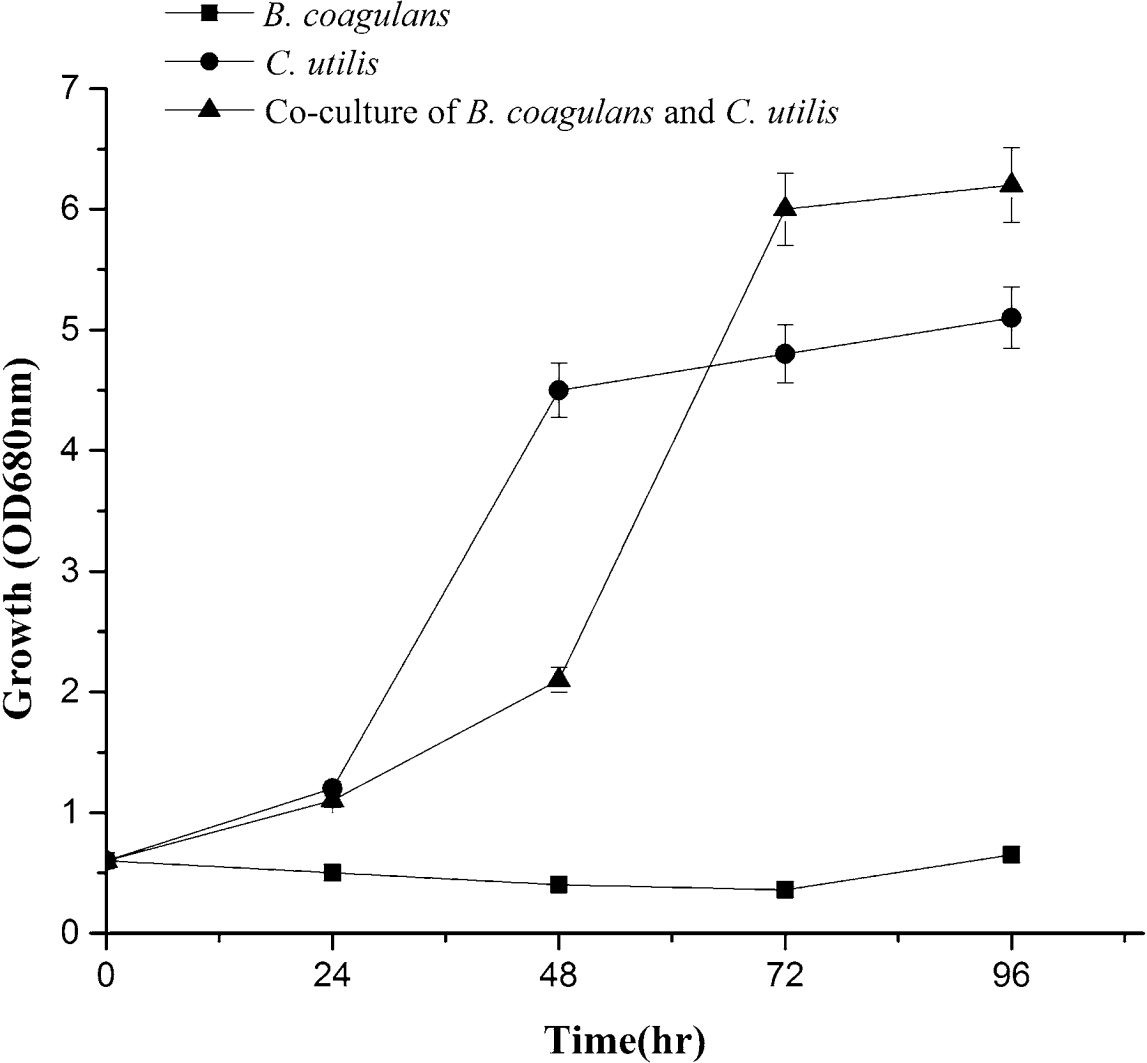
Growths of *B. coagulans* and *C. utilis* in single and co-culture using LFW as medium

**Fig. 3:**
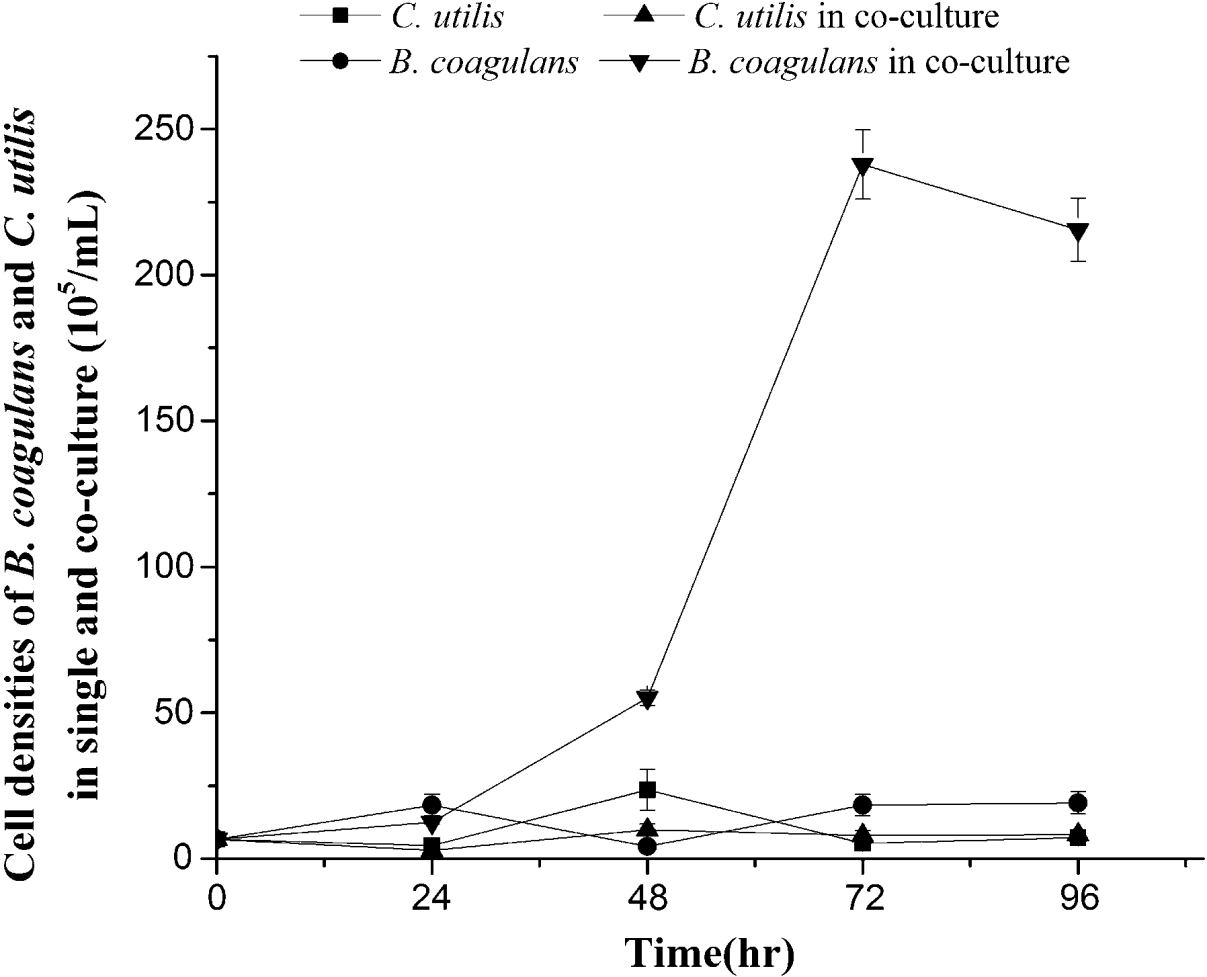
Cell densities of *B. coagulans* and *C. utilis* in single and co-culture using LFW as medium

Figure 2 showed the growth of total biomass (OD_680nm_) of *B. coagulans* and *C. utilis* using LFW as a culture medium, which indicated that *B. coagulans* could slightly grow but *C. utilis* grew relatively well in pure single culture. The maximum biomass was observed after day 3 in the presence of both *B. coagulans* and *C. utilis*, showing that, compared with those of single pure culture, the growth was highly enhanced in co-culture. Further investigation on the growth of two microbial strains in LFW tested by flow cytometer (Fig. 3) showed that the cell densities of *B. coagulans* and *C. utilis* were all increased from 6.56 × 10^5^ to 19.14 × 10^5^/mL and from 6.56 × 10^5^ to 7.26 × 10^5^/mL in the pure culture during the period of 4 days, respectively. However, in co-culture, both the growth of *C. utilis* (cell density from 6.56 × 10^5^ to 8.24 × 10^5^/mL) and especially *B. coagulans* (cell density from 6.56 × 10^5^ to 215.49 × 10^5^/mL) were much improved at the same time.

### Removal of lactic acid

**Fig. 4:**
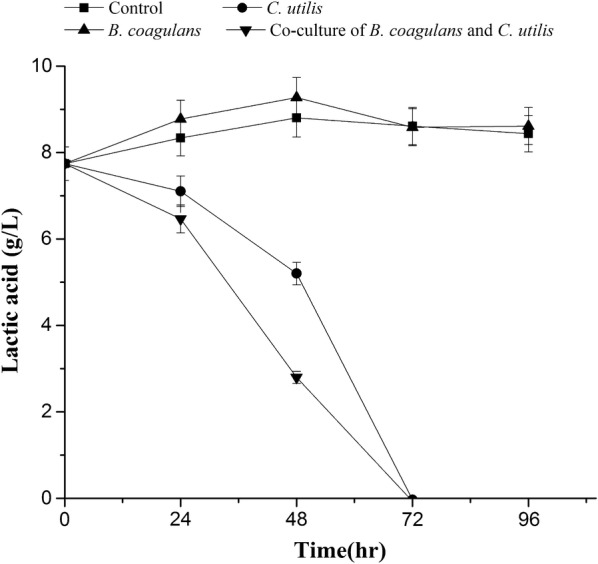
Removal of lactic acid from LFW by *B. coagulans* and *C. utilis*

Figure 4 showed the removal of lactic acid by *B. coagulans* and *C. utilis*, indicating that the concentrations of lactic acid was kept almost constant in control and slightly increased in the presence of *B. coagulans*, respectively, however initial lactic acid of 7.7 g/L was completed removed at 72 h by *C. utilis* both in single and co-culture, which showed that lactic acid could be produced by *B. coagulans* but removed by *C. utilis*, respectively.

### Removals of TOC and TN by *B. coagulans* and *C. utilis*

**Fig. 5:**
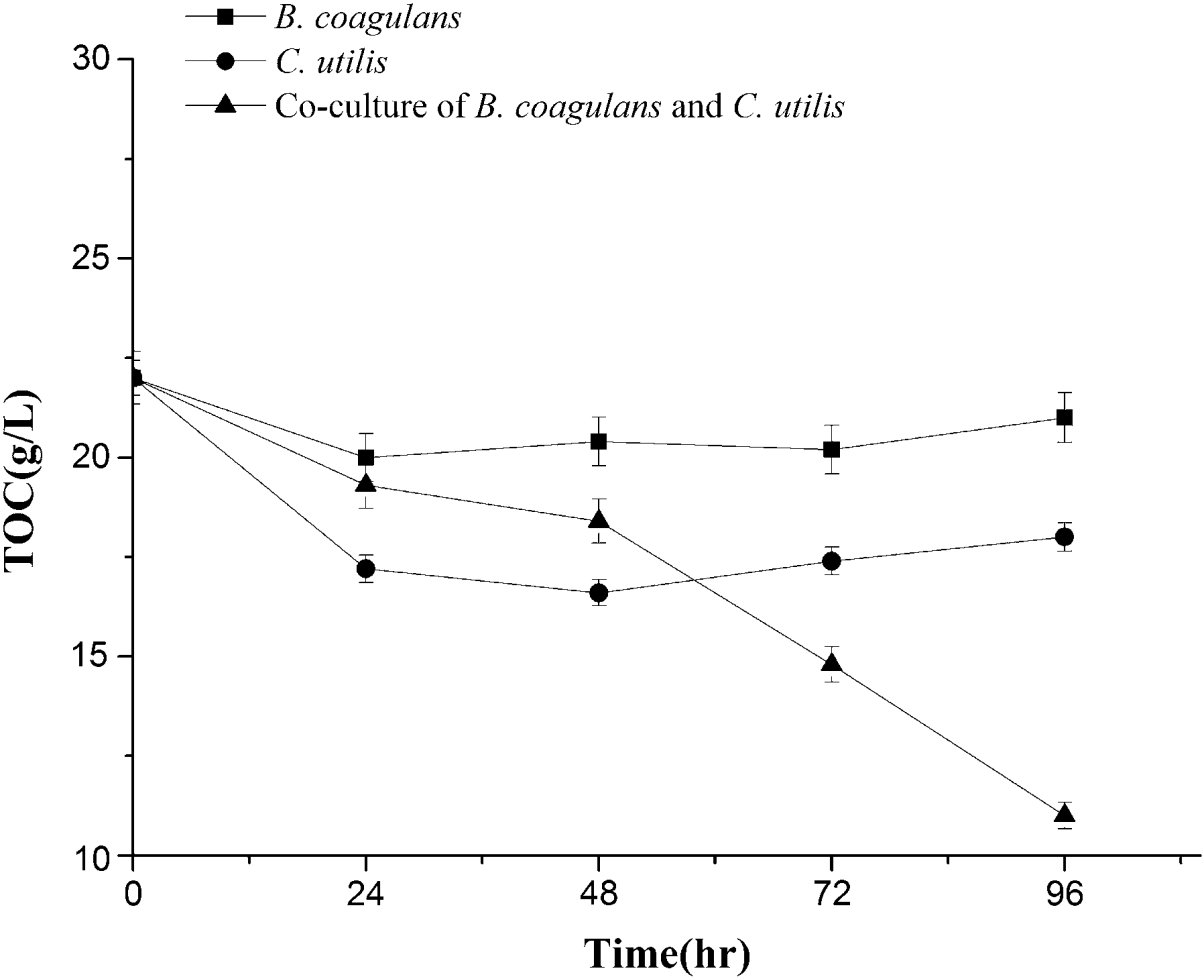
Removal of TOC by *B. coagulans* and *C. utilis* using LFW as medium

**Fig. 6:**
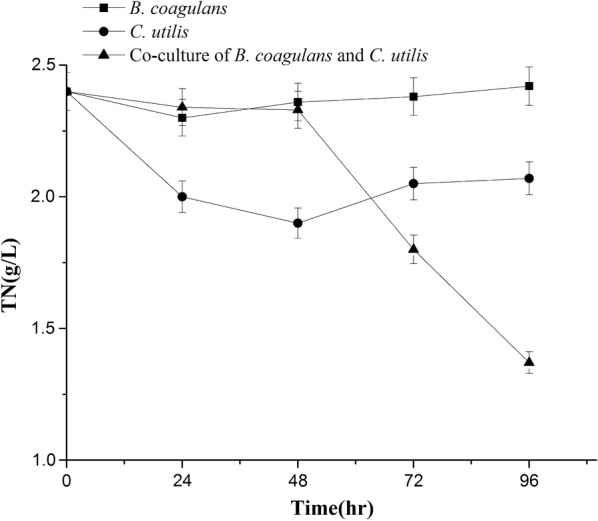
Removal of TN by *B. coagulans* and *C. utilis* using LFW as medium

**Fig. 7:**
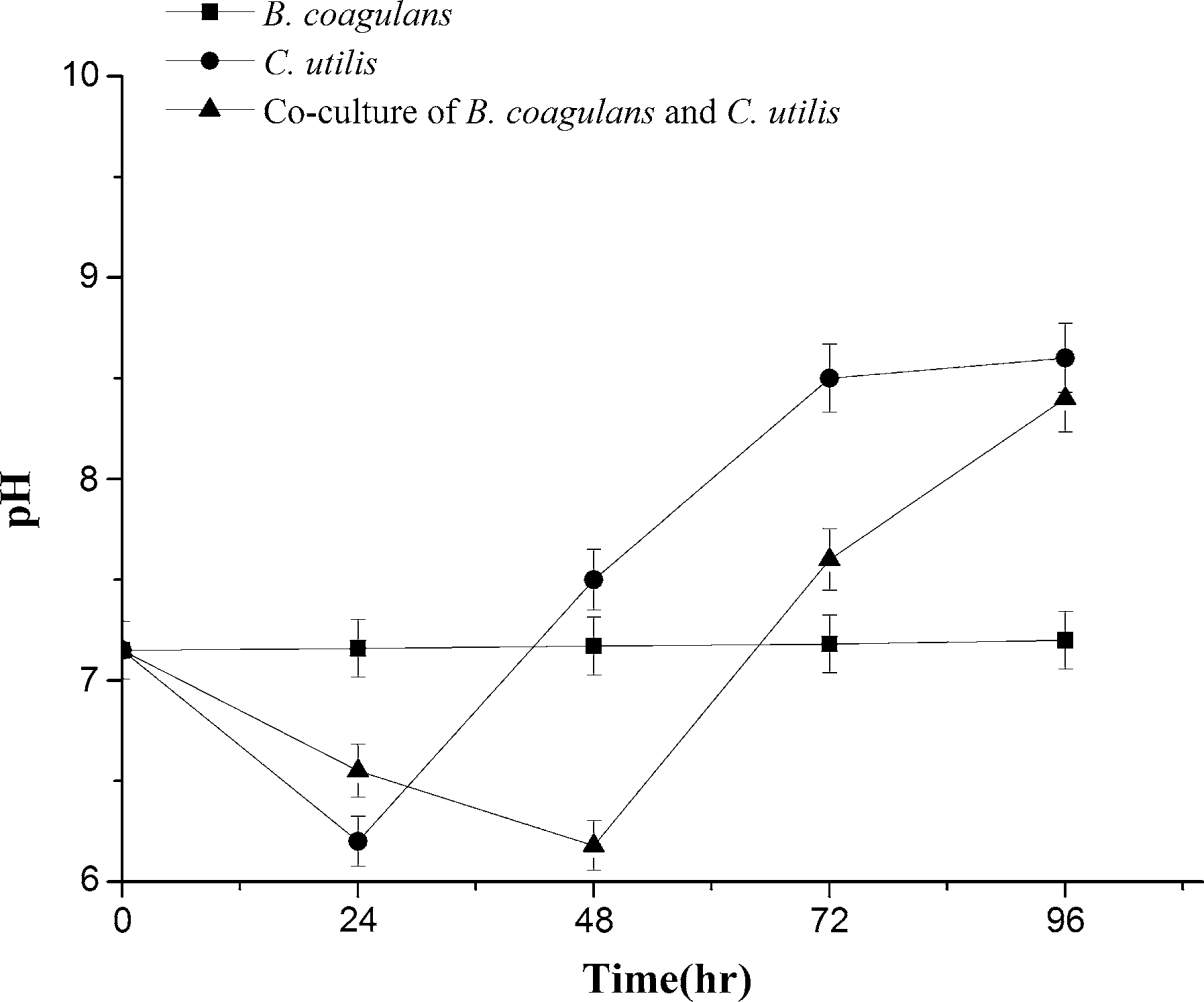
Changes of pH in the culture solution of *B. coagulans* and *C. utilis* using LFW as medium

As shown in Figs. 5 and 6, both TOC and TN declined slightly in single culture and apparently in co-culture. The removal ratios of TOC by *B. coagulans* and by *C. utilis* were 9.1% and 22.7%, respectively, which was improved to 49.0% in co-culture during the period of 96 h. The removal ratios of TN by *B. coagulans* and by *C. utilis* were 6.3% and 12.5%, which was also enhanced to 44.6% in co-culture at 96 h. The most efficiencies for the removals of both TOC and TN from LFW were achieved in the presence of *B. coagulans* and *C. utilis* simultaneously. Initial pH of 7.0 fluctuated from 6.0 to 9.0 during the period of 96 h (Fig. 7), which have not apparently adversely effects on the growth of *B. coagulans* and *C. utilis.*

## Discussion

With the increasing need for LAB, both their culturing scale and the production of LFW are rapidly growing, which is generally rich with organic compounds such as sugars and protein, as well as lactic acid produced by LAB (Table 1 and Fig. 4). The release of LFW into natural water body may cause cyanobacterial bloom that can produce toxins such as microcystins and nodularin (Wang et al. 2005), which threatens the safety of drinking water and human health. Because many nutrients such as sugar, protein and organic acids are remained in LFW (Table 1 and Fig. 4), which may waste the resource if it is simply treated by traditional processing. Unfortunately, the treatment technology of LFW, especially recycling it as a culture media for other probiotic strains, has not been reported in the literature up to now. So we firstly investigated the co-culture of two probiotic strains using LFW as a medium.

The size of the cells and their granularity, registered by a flow cytometer, may be used for separation of the mixture (Cruz and Bellakov 1996). Corzo et al. (1999) detected *Synechococcus* and *Prochlorococcus*-like populations by flow cytometry in a eutrophic reservoir. Here in the co-culture of *B. coagulans* and *C. utilis* in LFW, the cell densities of them could be measured with a flow cytometer (Fig. 1), respectively, which is very important in the identification and determination of the different microbial biomass in co-culture.

Generally, OD_680nm_ represents the total microbial biomass (Fig. 2), which cannot identify the cell densities of the different microbial strains. Here we successfully identified and measured the cell densities of both *B. coagulans* and *C. utilis* by flow cytometer (Fig. 3), respectively. Both *B. coagulans* and *C. utilis* are microbial probiotic strains and only pure culture rather than co-culture of them were reported up to now (Niu et al. 2011; Li and Liu 2006). When *B, coagulans* and *C utilis* were singly cultured in LFW, *C utilis* rather than *B. coagulans* grew well (Figs. 2, 3). However, in the presence of *C. utilis*, the growth of both especially *B. coagulans* was much improved (Figs. 2, 3). Firstly, the production of lactic acid may cause the effect of feedback inhibition on the growth of *B. coagulans*, but the metabolism of lactic acid by *C. utilis* can relieve the feedback inhibition to promote the growth of *B. coagulans* (Figs. 3, 4), which is a very important mechanism in co-culture. Oliva and Hang (1979) also found that *C. utilis* can grow and remove lactic acid from a continuous flow system of pickled wastewater. On another hand *C. utilis* might provide a number of nutritional factors such as amino acids and vitamins to enhance the growth of *B. coagulans.* Furthermore, the production of lactic acid from sugar remained in LFW by *B. coagulans* might also provide carbon source to enhance the growth of *C. utilis,* so the growths of both were improved each other in co-culture (Figs. 3, 4).

No information is provided on the treatment of LFW containing much amount of TOC and TN (Table 1), here we firstly investigated the resource utilization of LFW as a medium to culture other probiotic strains. The results indicated that the removals of both TOC and TN from LFW in co-culture of *B. coagulans* and *C. utilis* were much better than those of single culture (Figs. 5, 6). The previous studies also indicated that simultaneous removal of nutrients (ammonium and phosphate) and COD was better by the co-culture of *Chlorella vulgaris* and *Pseudomonas putida* than those of single culture, indicating that nutrients uptake capability of *C. vulgaris* was enhanced in the presence of *P. put*ida.

In summary, the flow cytometer was successfully used to identify and count the different cell densities of both *B. coagulans* and *C. utilis* (Fig. 1) using LFW containing much amount of TOC and TN (Table 1) as medium. Compared with *B. coagulans, C. utilis* relatively grew well in LFW in a single culture (Figs. 2, 3). The growths of two microbial strains especially *B. coagulans* were much improved in co-culture and the removal of lactic acid by *C. utilis* was responsible for the relief of feedback inhibition to increase the growth of *B. coagulans* (Figs. 2, 3 and 4). The promotion of microbial growth in co-culture was responsible for the efficient removals of TOC and TN (Figs. 5, 6), respectively, which came true the production of probiotics by the resource utilization of LFW as a medium.

## Abbreviations

LAB: lactic acid bacteria
LFW: lactobacillus fermentation wastewater
TOC: total organic carbon
TN: total nitrogen
TP: total phosphorus
